# Protective Effects of Allicin and Garlic-Derived Interventions Against Metabolic Dysfunction-Associated Steatotic Liver Disease: Current Evidence and Mechanistic Insights

**DOI:** 10.3390/nu18162596

**Published:** 2026-08-07

**Authors:** Niuniu Sun, Yongqiang Gao, Yu Xing, Mingyang Guo, Xinyu Xu, Jingxi Hu, Zitong He, Jiapeng Luo, Sheng Li, Zhaozhao Hui, Shunming Zhang

**Affiliations:** 1 School of Nursing, Henan University of Science and Technology, Luoyang 471003, China; 9905579@haust.edu.cn (N.S.); 18625901275@163.com (Y.G.); x2110677478@163.com (Y.X.); 13783097859@163.com (M.G.); 18238813861@163.com (J.L.); 17603790024@163.com (S.L.); 2 School of Public Health, Xi’an Jiaotong University Health Science Center, Xi’an 710061, China; xxyzhkdy@stu.xjtu.edu.cn (X.X.); hujingxi2028@163.com (J.H.); hezitong1020@163.com (Z.H.); huizzjoy@xjtu.edu.cn (Z.H.)

**Keywords:** allicin, garlic, non-alcoholic fatty liver disease, metabolic dysfunction-associated steatotic liver disease, review

## Abstract

Metabolic dysfunction-associated steatotic liver disease (MASLD) is the most prevalent chronic liver disease worldwide and poses a significant public health challenge. Allicin, a major bioactive organosulfur compound derived from garlic, has emerged as a promising candidate for MASLD prevention and management due to its antioxidant, anti-inflammatory, and metabolic regulatory properties. Despite its potential, evidence regarding allicin’s efficacy on MASLD remains limited and heterogeneous because existing studies have evaluated different garlic-derived interventions across diverse experimental settings. This review therefore distinguishes studies of purified allicin from those of raw garlic, garlic powder, aged garlic extract, black garlic, garlic essential oil, and individual garlic compounds. Preclinical studies using allicin suggest beneficial effects on hepatic steatosis, inflammation, glucose homeostasis, and lipid metabolism. Human studies, however, have evaluated raw garlic consumption or garlic powder supplementation rather than allicin alone. Findings from other garlic preparations and individual compounds are attributed only to the intervention tested, because differences in composition, stability, bioavailability, metabolism, and pharmacological activity preclude their attribution to allicin. Moreover, the available clinical trial evidence comprises five publications derived from only two randomized controlled trials conducted in Iran, both lasting 12–15 weeks. In addition, preclinical evidence most consistently supports regulation of hepatic lipid metabolism, particularly the AMP-activated protein kinase/sterol regulatory element-binding protein-1c-related signaling, as a potential mechanism underlying the anti-steatotic effects of allicin. Additional pathways may involve inflammatory signaling, oxidative stress, gut microbiota, and bile acid-related regulation, although the evidence for several of these mechanisms remains suggestive. Nevertheless, the precise molecular targets and signaling networks underlying these effects remain incompletely understood, and the relevance of allicin to aging-related MASLD is largely unexplored because previous studies have used young animals. Future studies should incorporate aged models and long-term randomized controlled trials across diverse populations to clarify the mechanisms, therapeutic potential, and safety of allicin in MASLD.

## 1. Introduction

Metabolic dysfunction-associated steatotic liver disease (MASLD), formerly known as non-alcoholic fatty liver disease, refers to excessive accumulation of fat in the liver, accompanied by at least one cardiometabolic risk factor (i.e., overweight/obesity, abnormal glucose metabolism, elevated blood pressure, elevated triglycerides, and reduced high-density lipoprotein cholesterol) [1]. MASLD ranges from simple steatosis to metabolic dysfunction-associated steatohepatitis, with potential progression to cirrhosis and hepatocellular carcinoma [2]. Affecting approximately 30–40% of the global adult population [3], MASLD is associated with an increased risk of cardiovascular disease, chronic kidney disease, and malignant tumors of the liver and extrahepatic sites [4]. Currently, extensive clinical trials that focus on MASLD resolution have emerged [5]. However, there are only two drugs (resmetirom and semaglutide) approved specifically for the treatment of non-cirrhotic metabolic dysfunction-associated steatohepatitis with moderate-to-advanced fibrosis (stages F2–F3) [6]. Importantly, the therapeutic benefits of these medications remain limited, as only a proportion of patients achieve histological improvement, and their efficacy outside the F2–F3 fibrosis stage remains uncertain [6]. Therefore, there remains an urgent need to explore additional therapeutic strategies for MASLD.

In recent years, dietary bioactive compounds have attracted increasing interest as potential approaches to support MASLD management [7]. For example, a recent randomized controlled trial (RCT) reported that grape seed extract supplementation improved several markers of oxidative stress in patients with MASLD, although further confirmation is required [8]. In addition, garlic is widely consumed and contains several bioactive organosulfur compounds. Among these compounds, allicin has attracted particular interest because of its potential effects on lipid metabolism, oxidative stress, and inflammation, which are closely involved in the development and progression of MASLD [9,10]. Recent experimental evidence indicates that allicin reduces hepatic lipid accumulation and improves metabolic disturbances in mice with diet-induced steatohepatitis, accompanied by modulation of the circadian clock gene Rev-erbα [11]. Moreover, as MASLD progresses from steatosis to fibrosis and cirrhosis, oxidative stress and inflammation play critical roles in this pathological progression [9]. Consistent with its anti-inflammatory potential, allicin at 40 mg/kg significantly reduced serum tumor necrosis factor-α, interleukin-1β, and interleukin-6 concentrations in a carbon tetrachloride-induced acute liver injury model [12]. Although this model differs from MASLD, the findings provide supportive evidence of the anti-inflammatory activity of allicin. Furthermore, the progression and therapeutic effect of MASLD can be influenced by the regulation of the composition and function of the gut microbiota [10]. Allicin inhibits the enterohepatic fibroblast growth factor 15-7α-hydroxylase 1 signaling through modulation of the gut microbiota and bile acid profile, thereby promoting bile acid synthesis and reducing hepatic cholesterol levels [13]. Taken together, allicin has demonstrated promising efficacy in preventing and ameliorating MASLD. Additionally, a cross-sectional study in China reported that raw garlic consumption was inversely associated with the prevalence of MASLD in males but not in females [14]. This finding provides initial population-based evidence supporting a beneficial role of garlic consumption (allicin as the main active component) in MASLD, while the underlying mechanisms and sex-specific patterns warrant further investigation.

Previous reviews have summarized the broad biological activities of allicin and garlic-derived compounds, while systematic reviews and meta-analyses have mainly evaluated the effects of garlic supplementation on selected clinical or preclinical outcomes in MASLD [15,16,17]. In contrast, the present mini-review provides an updated synthesis of population, clinical, animal, and cell-based evidence within the current MASLD framework. Particular attention is given to distinguishing direct evidence obtained using isolated allicin from supportive evidence involving garlic preparations or related organosulfur compounds. In addition, this review integrates the potential mechanisms underlying these effects and highlights important translational gaps, including heterogeneous interventions, limited clinical populations, and the lack of aged experimental models.

## 2. Source and Metabolism of Allicin

**Figure 1:**
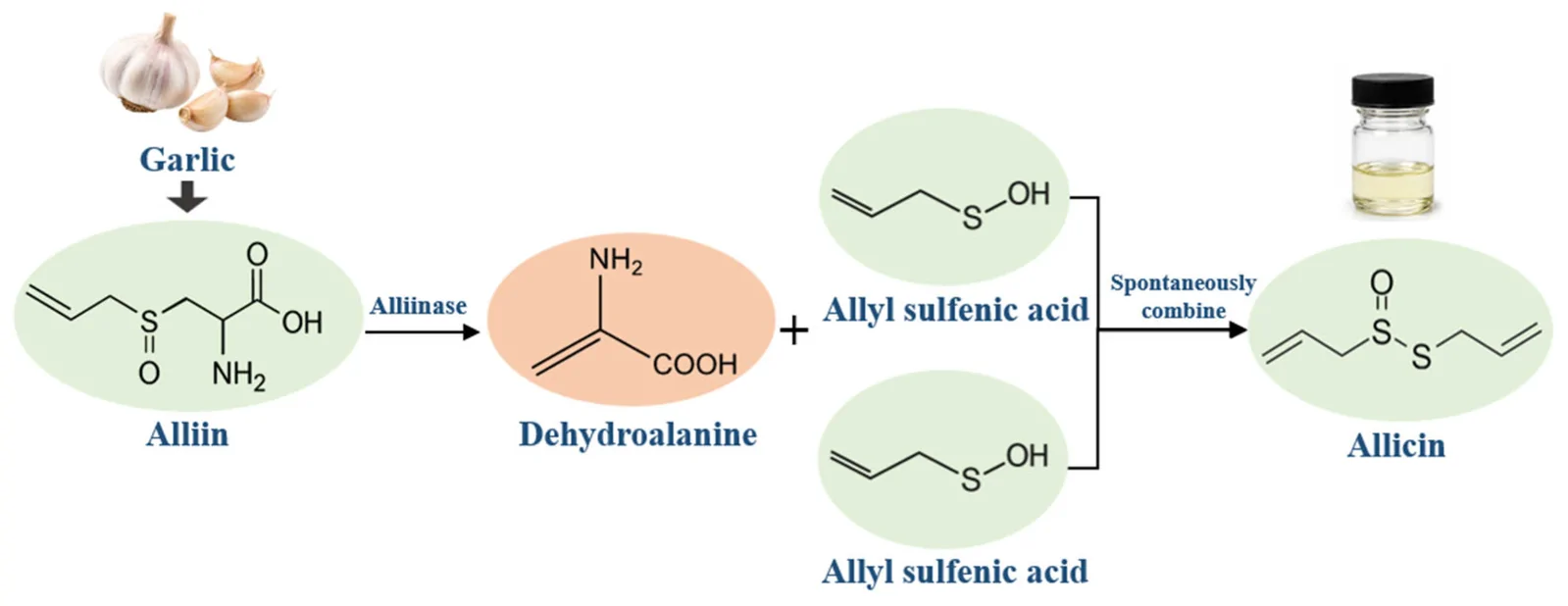
The source of allicin.

Garlic has been valued for thousands of years and was considered one of the most precious foods and medicinal preparations in ancient India and China [18]. Allicin was first discovered in 1944, when Cavallito and Bailey isolated it from garlic and provided a detailed account of its preparation method, physicochemical properties, and notable antibacterial activity. This landmark finding marked the beginning of modern allicin research [19]. Allicin is a thiosulfinate, and its structure was determined by Stoll and Seebeck in 1948 [20]. By 1951, Stoll and Seebeck had elucidated the structure of alliin as its precursor, along with the process by which alliinase catalyzes its conversion into allicin, thereby fully revealing the “alliin–alliinase” system [18]. When raw garlic is chewed or crushed, disruption of the garlic tissue brings alliin into contact with alliinase, which catalyzes its conversion into allyl sulfenic acid and dehy-droalanine. Whereas dehydroalanine is further converted to pyruvate and ammonia, two molecules of allyl sulfenic acid spontaneously combine to form one molecule of allicin (Figure 1) [15,21].

It is noteworthy that allicin undergoes remarkable chemical transformations upon entering the human body. However, very few reports of accurate methods for direct pharmacokinetic studies of allicin in human plasma exist because of its decomposition rate. The main metabolic pathway is that after ingesting allicin, it is rapidly broken down in the gastrointestinal tract, generating many secondary degradation compounds, such as sulfur dioxide, diallyl sulfide, diallyl trisulfide, diallyl disulfide, and ajoene in the presence of enzymatic reactions in gastric juice [22,23]. Due to the significant first-pass effect, its metabolism rate is extremely rapid [21]. The liver is the main organ for the metabolism of allicin. Meanwhile, reduction, methylation, and oxidation are the main metabolic reactions of allicin. Allicin is rapidly decomposed in the liver to form a variety of organic sulfides, including S-allylmercaptoglutathione, S-allylmercaptocysteine, allyl methyl sulfide, and allyl mercaptan [10]. Subsequently, it is mainly excreted through urine and exhalation [24].

## 3. Materials and Methods

To provide a focused overview, a narrative literature search was conducted in PubMed and Web of Science from database inception to 30 June 2026. The search combined the terms “allicin” and “garlic” with “MASLD” and its earlier terminology, including “non-alcoholic fatty liver disease” (“NAFLD”) and “metabolic-associated fatty liver disease” (“MAFLD”). Original observational studies, RCTs, animal experiments, and in vitro studies were included if they investigated allicin or garlic-derived preparations and reported outcomes related to MASLD, such as hepatic steatosis, liver injury, glucose or lipid metabolism, oxidative stress, inflammation, or gut microbiota-related changes. In addition, reviews, meta-analyses, editorials, conference abstracts, duplicate publications without additional relevant data, studies concerning liver diseases unrelated to MASLD, and studies without relevant hepatic or metabolic outcomes were excluded. The reference lists of relevant articles were also manually screened, and all studies identified through this search that met the eligibility criteria were included. For evidence synthesis, the interventions were classified into three categories: (1) isolated allicin or allicin-specific delivery formulations; (2) complex garlic preparations, including garlic powder, aged garlic extract, black garlic, garlic essential oil, and garlic-derived nanoparticles; and (3) other defined garlic-derived compounds, including alliin, diallyl disulfide, S-allyl cysteine, and S-allylmercaptocysteine. Findings from the latter two categories were interpreted as preparation- or compound-specific supportive evidence and were not attributed directly to allicin.

## 4. Evidence for Allicin and Other Garlic-Derived Interventions in MASLD

### 4.1. Observational Studies

A cross-sectional study conducted in China reported an inverse association between raw garlic consumption and the prevalence of MASLD in males, whereas no significant association was observed in females [14]. After adjustment for age, body mass index, sociodemographic characteristics, lifestyle factors, total energy consumption, overall dietary patterns, and onion consumption, each 1 g/1000 kcal increment in daily raw garlic consumption was associated with an odds ratio (OR) of 0.93 (95% confidence interval [CI]: 0.90–0.97) in males and 1.05 (95% CI: 0.98–1.11) in females. This sex disparity may partly be explained by differences in estrogen status, body fat distribution, dietary behaviors, and metabolic profiles between males and females.

Overall, the current observational evidence remains very limited, as only one cross-sectional study has investigated the association between raw garlic consumption and MASLD. Although the study adjusted for demographic, lifestyle, and dietary factors, the cross-sectional design precluded causal inference and could not establish the temporal relationship between raw garlic consumption and MASLD. In addition, the available population-based studies have predominantly been conducted in Asian populations [25]. Therefore, large-scale prospective cohort studies with long-term follow-up are warranted to determine whether higher raw garlic consumption is associated with a reduced risk of incident MASLD and whether such association differs between male and female populations. Future epidemiological studies should also investigate the biological mechanisms underlying any observed associations. Multi-omics studies in metabolic dysfunction-associated steatohepatitis have demonstrated the value of integrating microbiome, metabolomic, and immunological data to characterize gut–liver interactions [26]. Applying similar approaches to future studies of garlic consumption may help identify potential pathways involving lipid metabolism, inflammation, and microbiota–host interactions.

### 4.2. RCTs

Five publications derived from two independent RCTs have evaluated garlic powder supplementation in patients with MASLD [27,28,29,30,31]. Three publications by Soleimani et al. reported different outcomes from the same 15-week trial, in which 110 patients were randomized and 98 completed the intervention [27,28,31]. The two publications by Sangouni et al. similarly reported different outcomes from the same 12-week trial, in which 90 patients were randomized and 88 completed the study [29,30]. Thus, the five publications represent two independent clinical cohorts rather than five separate confirmations of efficacy. The characteristics of these five publications are summarized in Table 1. Although favorable changes were reported in hepatic steatosis and several metabolic outcomes, both trials evaluated garlic powder rather than purified allicin.

Importantly, garlic powder is a complex mixture containing multiple bioactive compounds rather than a purified form of allicin. Its biological effects may reflect the action of allicin generated from alliin by alliinase together with other sulfur-containing constituents [15,16]. Allicin may therefore be a major contributor to the biological activity of garlic powder when alliinase activity is preserved, although its predominant role has not been directly established. However, the included studies reported only the nominal allicin content and did not provide comprehensive chemical profiling of the tested products. Potential additive or synergistic effects among garlic constituents are therefore plausible but were not directly evaluated. Accordingly, the clinical findings should be attributed to the specific garlic powder preparations studied rather than to allicin alone.

Garlic powder products may differ substantially in their chemical composition. The contents of alliin, allicin-forming capacity, and other organosulfur compounds may be influenced by garlic cultivar, geographical and agroecological conditions, sulfur nutrition, drying procedures, and storage conditions. However, published studies from different regions have not always analyzed the same form of garlic: some evaluated finished garlic powder supplements, whereas others analyzed experimentally prepared powders or garlic materials relevant to powder production. Therefore, the material and processing method evaluated in each study are specified in Table 2. The studies included in this table are intended to provide representative compositional context rather than an exhaustive global survey.

As shown in Table 2, the organosulfur composition of garlic materials varies substantially across geographical regions, cultivars, and processing conditions. However, geographical origin cannot be considered an independent determinant of composition because cultivar, sulfur nutrition, growing conditions, harvest maturity, drying temperature, storage, sample preparation, and analytical methodology may all contribute to the observed variation. In particular, intact garlic powder primarily contains alliin and alliinase rather than stable preformed allicin, and its actual allicin-forming capacity depends on the preservation and activation of alliinase. Garlic powder also contains other thiosulfinates, γ-glutamyl peptides, and non-sulfur constituents that may exert additive or synergistic effects. Nevertheless, such interactions have rarely been directly evaluated. Therefore, the regional compositional data summarized in these studies provide useful background for considering potential contributors to heterogeneity among studies but cannot be used to infer the exact composition or bioavailability of the garlic powder products administered in the clinical trials summarized in Table 1. The reported clinical effects should consequently be attributed to the tested garlic powder preparations rather than to allicin alone.

Taken together, evidence from two independent RCTs suggests that garlic powder supplementation for 12–15 weeks may improve ultrasonography-detected hepatic steatosis and selected metabolic outcomes in patients with MASLD. However, the five publications were derived from only two clinical cohorts and should not be interpreted as five independent confirmations of efficacy. Both trials were conducted in Iran, included relatively modest samples, and reported predominantly favorable outcomes over short intervention periods, which limits the generalizability of their findings and means that selective outcome reporting and publication bias cannot be excluded. Hepatic steatosis was assessed using conventional ultrasonography in both trials, while neither liver biopsy nor liver elastography was performed. Conventional ultrasonography has limited sensitivity for detecting longitudinal changes and cannot determine whether garlic powder improves steatohepatitis or liver fibrosis. Although liver biopsy provides histological information, its invasive nature makes its routine use difficult to justify in short-term nutritional intervention trials. Future studies should therefore incorporate quantitative, non-invasive assessments, particularly vibration-controlled transient elastography, with liver stiffness measurement used to assess fibrosis and the controlled attenuation parameter used to assess hepatic steatosis [39]. Reporting of adverse events was limited, and long-term safety remains uncertain. Furthermore, geographical, cultivar-related, and processing-related variability in garlic composition, together with incomplete batch-specific characterization and the absence of allicin bioavailability measurements, limits comparisons across studies and prevents the observed effects from being attributed specifically to allicin. A previous meta-analysis reported improvements in hepatic steatosis and selected metabolic outcomes following garlic supplementation; however, its pooled estimates were derived from the same two independent clinical trials discussed above and therefore provide limited additional certainty beyond the primary studies [17]. The trials used fixed daily doses rather than body-weight-adjusted doses. Garlic powder was administered at 800–1600 mg/day, corresponding to a reported allicin dose of 3–6 mg/day, for 12–15 weeks [27,28,29,30,31]. However, because no dose-ranging or duration-comparison studies have been conducted, the optimal dose per kilogram of body weight and the optimal duration of treatment remain unclear. Accordingly, the current evidence is insufficient to recommend a body-weight-adjusted amount of fresh garlic, garlic powder, or garlic extract, and this question should be addressed in future dose-ranging trials. Larger, long-term, multicenter trials involving diverse populations, standardized and chemically characterized preparations, multiple-dose groups, systematic safety assessment, and more accurate evaluation of hepatic inflammation and fibrosis are required.

In addition, none of the available RCTs reported treatment effects stratified by sex, age, or comorbidity status. As summarized in Table 1, the reported mean ages across the trial groups ranged from 44.1 to 46.4 years. It remains unclear whether garlic powder has comparable effects in males and females, younger and older adults, or patients with common metabolic comorbidities. Potential differences between males and females should therefore be considered in future studies. Future trials should adopt a precision nutrition approach by including more diverse populations and conducting prespecified analyses according to sex, age, metabolic phenotype, and comorbidity status. This approach may help identify individuals who are most likely to benefit and support more individualized recommendations. Where feasible, exploratory studies involving monozygotic twin pairs may help assess interindividual variability while partially controlling for genetic background, although their recruitment and implementation would be challenging.

### 4.3. Animal Experiments

Animal experiments evaluating the effects of garlic-derived interventions on hepatic and metabolic outcomes related to MASLD were published between 2013 and 2026 [11,13,40,41,42,43,44,45,46,47,48,49,50,51,52]. The detailed characteristics of these studies are summarized in Table 3.

Among the included animal studies, the most direct evidence was provided by experiments that administered isolated allicin, either alone or through a defined delivery system [11,13,40,47]. These studies reported improvements in hepatic steatosis, inflammation, glucose and lipid metabolism, and gut microbiota-related parameters. Nevertheless, differences in disease models, allicin formulations, dosages, administration routes, and treatment durations limit direct comparisons among these studies.

Other studies evaluated complex garlic-derived preparations, including aged garlic extract, black garlic, garlic essential oil, garlic-derived exosome-like nanoparticles, and a synbiotic containing aged garlic extract [41,42,44,45,46,49]. Because these preparations contain multiple constituents and their chemical profiles vary according to processing, storage, and formulation, their biological effects cannot be attributed specifically to allicin. These findings instead provide preparation-specific evidence for the broader potential of garlic-derived interventions in MASLD models. Moreover, most of these preparations were not comprehensively chemically characterized, which limits comparisons across studies and prevents identification of the constituents responsible for the observed effects.

Evidence also indicates that several chemically defined garlic constituents other than allicin have biological activity in models of MASLD. Alliin, the precursor of allicin, reduced endoplasmic reticulum (ER) stress, hepatic steatosis, and lipid accumulation in tunicamycin-treated HepG2 cells and mice [43]. Diallyl disulfide, administered either as an isolated compound or as a major constituent of garlic essential oil, ameliorated obesity-associated hepatic steatosis, dyslipidemia, and oxidative stress in high-fat diet-fed mice [49]. S-allyl cysteine improved glycemic control and hepatic lipid metabolism and reduced steatosis in diabetic OLETF rats [51]. S-allylmercaptocysteine reduced hepatic lipid accumulation, liver injury, apoptosis, fibrosis, and oxidative stress and was also associated with enhanced autophagy in rat models of MASLD [50,52]. In addition, the non-sulfur garlic-skin antioxidants *N*-trans-p-coumaroyloctopamine and *N*-trans-feruloyloctopamine attenuated oxidative stress and liver injury in rats with steatohepatitis [48]. These findings demonstrate that the biological potential of garlic is not restricted to allicin and suggest that other constituents may contribute to the effects of complex garlic preparations. However, because these compounds differ from allicin in structure, stability, metabolism, and pharmacological activity, their effects should be interpreted as evidence specific to each compound rather than as evidence for allicin. Moreover, their contribution to the clinical effects of garlic powder remains uncertain because the included RCTs did not report their concentrations in the tested products. Notably, most studies were conducted in young mice or rats (typically 3–6 months old), whereas aged models (≥18 months) remain largely underexplored. Consequently, the potential effects of allicin on aging-related MASLD cannot be adequately assessed based on the current evidence.

Overall, the available animal evidence supports the potential hepatoprotective effects of several garlic-derived interventions, but the findings are intervention-specific and should not be considered pharmacologically equivalent. Conclusions concerning allicin are therefore based primarily on studies that directly administered isolated allicin, either alone or through a defined delivery system. Interpretation is further limited by heterogeneity in animal species, disease models, dosage, administration route, treatment duration, age, and sex. The incomplete chemical characterization of several complex preparations represents an additional limitation and precludes direct comparison of their pharmacological effects with those of isolated allicin. Although the animal studies used body-weight-adjusted doses, these doses cannot be directly extrapolated to humans because of differences in species, garlic preparations, routes of administration, and bioavailability.

### 4.4. Cell Experiments

Previous cell experiments have demonstrated that allicin reduces lipid accumulation and regulates lipid metabolism [53]. Pretreating foam cells with allicin was found to reduce lipid accumulation, including levels of total cholesterol, free cholesterol, and cholesterol esters. Additionally, allicin upregulates the expression of ATP-binding cassette transporter A1, which enhances cholesterol efflux through the peroxisome proliferator-activated receptor γ (PPARγ)/liver X receptor α signaling pathway in foam cells. Another study investigated the protein targets of allicin on lipid metabolism [54]. Allicin was found to upregulate the expression of peroxisome proliferator-activated receptor α and fatty acid-binding protein 6, while downregulating the expression of fatty acid-binding protein 4 and PPARγ. In 1,3-Dichloro-2-propanol-induced HepG2 cells, allicin alleviated lipid accumulation and disorders in lipid metabolism by modulating both the AMP-activated protein kinase (AMPK)/sterol regulatory element-binding protein-1c (SREBP-1c) and the protein kinase A/cAMP-response element-binding protein signaling pathways [55]. The cellular studies discussed above directly evaluated allicin. Studies investigating alliin, diallyl disulfide, S-allyl cysteine, S-allylmercaptocysteine, or other garlic-derived compounds were considered separately because these compounds differ from allicin in chemical structure, stability, metabolism, and pharmacological properties and cannot be regarded as functional substitutes for allicin. The concentrations of allicin used in vitro varied considerably across experimental models, which limits direct comparisons among studies and complicates translation of the findings to in vivo exposure. While these preclinical findings are promising, they are based on specific, induced pathologies, and the preventive or therapeutic window in humans remains undefined. For example, the use of a cancer cell line to model metabolic disease is a limitation, as the results may not fully replicate physiology in normal hepatocytes.

## 5. Molecular Mechanisms Underlying the Protective Effects of Allicin Against MASLD

Allicin may ameliorate MASLD through the coordinated modulation of multiple pathogenic processes, including hepatic lipid accumulation, insulin resistance, oxidative stress, inflammatory activation, and gut microbiota and bile acid-related alterations [9,11,13,40,47,55]. However, the strength and directness of evidence differ among these proposed mechanisms. In this section, findings obtained using isolated allicin in MASLD-relevant animal or hepatic cellular models are considered direct evidence. Findings derived from complex garlic preparations, related organosulfur compounds, non-hepatic cells, or non-MASLD disease models are considered indirect or supportive evidence. Mechanisms that have not been directly examined following allicin administration are presented as hypotheses. Among the proposed pathways, suppression of hepatic de novo lipogenesis, particularly through AMPK/SREBP-1c-related signaling, currently has the strongest direct preclinical support [40,55].

The most consistently supported potential mechanism underlying the effects of allicin is the regulation of hepatic lipid metabolism. Excessive activation of sterol regulatory element-binding proteins, particularly SREBP-1c, promotes hepatic lipogenesis and triglyceride accumulation. In cellular models of lipid metabolic disorder, allicin alleviates intracellular lipid accumulation through modulation of both the AMPK/SREBP-1c and protein kinase A/cAMP-response element binding protein signaling pathways [55], thereby suppressing lipogenesis while restoring metabolic balance. Consistently, allicin has been shown to upregulate peroxisome proliferator-activated receptor α and fatty acid-binding protein 6, while downregulating PPARγ and fatty acid-binding protein 4, indicating a shift from lipid storage toward lipid utilization [54]. This is further supported by recent experimental evidence showing that allicin significantly suppresses the expression of key lipogenic genes, including acetyl-CoA carboxylase, fatty acid synthase, stearoyl-CoA desaturase-1, and SREBP-1c, thereby reducing hepatic lipid accumulation in both in vitro and in vivo models [40]. These findings collectively suggest that suppression of hepatic de novo lipogenesis contributes importantly to the protective effects of allicin against MASLD. Nevertheless, this conclusion is based predominantly on preclinical evidence, and the involvement of this pathway has not been confirmed in patients receiving purified allicin.

Indirect evidence from non-hepatic foam-cell models suggests that allicin may enhance cholesterol efflux through the PPARγ/liver X receptor α/ATP-binding cassette transporter A1 pathway [53]. Although this finding may be relevant to cholesterol-induced lipotoxicity, it has not been directly demonstrated in hepatocytes or MASLD models. Therefore, cholesterol efflux should currently be regarded as a supportive rather than established hepatic mechanism of allicin.

Lipophagy mediates the delivery of intracellular lipid droplets to lysosomes for degradation and is impaired during MASLD progression. This impairment may occur at several stages of the autophagy–lysosome pathway, including reduced autophagy initiation and autophagosome formation associated with dysregulated AMPK–mTORC1–ULK1 signaling, defective autophagosome–lysosome fusion, and impaired lysosomal acidification and lipid degradation. Chronic nutrient overload, lipotoxicity, and oxidative stress may further aggravate these defects [56,57,58]. However, none of the included studies directly examined whether allicin affects autophagic flux, lysosomal activity, or lipophagic degradation in a MASLD model. Therefore, the involvement of lipophagy in the effects of allicin remains hypothetical and requires direct experimental investigation.

Allicin also exerts systemic metabolic benefits by improving insulin sensitivity. Insulin resistance is a defining feature of MASLD and contributes to hepatic steatosis through increased adipose lipolysis, elevated free fatty acid flux, and enhanced hepatic lipogenesis. RCTs have shown that garlic supplementation improves fasting glucose, insulin levels, and insulin resistance, along with reductions in body fat and waist circumference in patients with MASLD [29]. These clinical findings indicate that garlic powder supplementation may improve systemic metabolic dysfunction in patients with MASLD. However, because the available RCTs evaluated garlic powder rather than purified allicin, they cannot establish that the improvements in insulin resistance were specifically mediated by allicin.

Oxidative stress represents an additional potential target of garlic-derived interventions in MASLD [59,60]. ER stress refers to disruption of protein-folding homeostasis within the ER, resulting in the accumulation of unfolded or misfolded proteins and activation of the unfolded protein response. This response is mediated mainly by inositol-requiring enzyme 1 alpha, protein kinase RNA-like ER kinase, and activating transcription factor 6. Persistent activation of these pathways can disturb hepatic lipid metabolism and promote inflammatory and apoptotic signaling. In the included study, tunicamycin increased the expression of inositol-requiring enzyme 1, protein kinase RNA-like ER kinase, activating transcription factor 6, glucose-regulated protein 78, CCAAT/enhancer-binding protein homologous protein, and X-box-binding protein 1, whereas alliin attenuated these changes together with hepatic lipid accumulation [43]. However, this evidence concerns alliin rather than isolated allicin, and no included study directly examined the effects of allicin on ER stress signaling in a MASLD model. Therefore, regulation of ER stress remains a proposed rather than demonstrated mechanism of allicin.

Gut–liver axis dysfunction is an important contributor to MASLD progression [61,62]. Deng et al. provided direct evidence that allicin supplementation ameliorated hepatic inflammation and gut microbiota dysbiosis in high-fat, high-fructose diet-induced NASH mice [11]. Allicin also partially restored alterations in the hepatic circadian clock gene Rev-erbα, and knockdown of Rev-erbα attenuated its anti-inflammatory effects in HepG2 cells, supporting the involvement of this pathway. Additional studies in diabetic rats and high-fat diet-induced obese mice showed that allicin altered gut microbiota composition, bile acid profiles, or related metabolic outcomes [13,47], although these models were not specifically designed to reproduce MASLD. Collectively, the available evidence demonstrates that allicin can modify gut microbiota-related parameters. However, whether these microbial alterations causally mediate its hepatic effects remains uncertain because microbiota depletion, fecal microbiota transplantation, or germ-free experiments have not been performed.

Beyond the direct regulation of hepatic lipid metabolism and inflammation, emerging evidence from non-MASLD models suggests that garlic-derived sulfur compounds may also influence aging-related metabolic pathways. In aged mice, S-1-propenyl-L-cysteine, a sulfur-containing constituent of aged garlic extract, activated the liver kinase B1-sirtuin 1 pathway, enhanced extracellular nicotinamide phosphoribosyltransferase secretion, and improved frailty-related phenotypes through hypothalamic signaling [63]. Although these findings do not involve allicin or MASLD, they suggest that aging-related metabolic signaling may represent an additional mechanistic context for garlic-derived sulfur compounds. Given that most MASLD studies of allicin have used young adult animals, whether similar pathways contribute to the effects of allicin in aging-related MASLD remains to be determined.

Taken together, the proposed mechanisms of allicin in MASLD are supported by different levels of evidence. Suppression of hepatic de novo lipogenesis, particularly through AMPK/SREBP-1c-related signaling, currently has the strongest direct preclinical support. The involvement of Rev-erbα in the anti-inflammatory effects of allicin has also received direct experimental support, whereas the causal contribution of gut microbiota alterations remains uncertain. Evidence concerning insulin resistance, cholesterol efflux, oxidative stress, and ER stress is partly derived from garlic preparations, non-hepatic cells, or non-MASLD models and should therefore be considered supportive. Lipophagy and aging-related signaling remain hypothetical. Further studies using purified allicin, MASLD-specific models, and pathway-targeted experiments are required to establish causality.

## 6. Conclusions

Preclinical studies suggest that allicin may exert protective effects in MASLD models, with suppression of hepatic de novo lipogenesis through AMPK/SREBP-1c-related signaling representing the most consistently supported potential mechanism. However, the available human studies evaluated raw garlic consumption or garlic powder rather than allicin alone, although allicin is among their major bioactive constituents. Therefore, the clinical efficacy and safety of purified allicin in MASLD remain unknown. Whether the clinical effects of garlic-derived interventions differ according to sex, age, or comorbidity status also remains unclear. Geographical, cultivar-related, and processing-related variability in garlic composition further limits comparisons among garlic powder studies and emphasizes the need for batch-specific chemical characterization of future clinical interventions. Defined garlic constituents other than allicin, including alliin, diallyl disulfide, S-allyl cysteine, and S-allylmercaptocysteine, have also shown beneficial effects in preclinical models of MASLD, suggesting that these compounds may contribute to the biological activity of complex garlic preparations. However, their contribution to the effects observed in clinical studies remains uncertain because the garlic powder products were not comprehensively characterized. Evidence obtained using aged garlic extract, black garlic, garlic essential oil, and other garlic-derived preparations should be interpreted as preparation- or compound-specific supportive evidence rather than as direct evidence for allicin. Moreover, the relevance of allicin to aging-related MASLD remains largely unexplored, given that most experimental studies have been conducted in young mice. Future studies should therefore prioritize standardized allicin formulations, utilize aged animal models, and conduct well-designed long-term RCTs to more thoroughly define the efficacy, mechanisms, and translational potential of allicin in MASLD.

## Figures and Tables

**Table 1:** Characteristics of five publications derived from two double-blind, placebo-controlled randomized trials of garlic powder in MASLD.

References	Number of Participants	Age (Years)		Sex		Duration (Week)	T	Composition	Main Findings
		Mean ± SD		Male/Female					
		T	C	T	C				
Soleimani et al. (2016) [27]	98	46.4 ± 11.3	44.1 ± 11.8	19/28	21/30	15	Garlic powder, 400 mg/tablet, twice daily (800 mg/day), vs. placebo	Reported allicin: 1.5 mg/tablet (3 mg/day). Other organosulfur constituents: NR.	After 15 weeks of garlic supplementation, patients had a significant loss in body weight and body fat mass.
Soleimani et al. (2020) [28]	98	NR	NR	NR	NR	15	Garlic powder, 400 mg/tablet, twice daily (800 mg/day), vs. placebo	Reported allicin: 1.5 mg/tablet (3 mg/day). Other organosulfur constituents: NR.	Patients in the intervention group achieved significant improvement in hepatic steatosis, liver enzymes, and lipid profile compared to placebo.
Sangouni et al. (2020a) [29]	88	45.2 ± 12.4	44.2 ± 11.1	33/12	24/19	12	Garlic powder, 400 mg/tablet, four tablets/day (1.6 g/day), vs. placebo	Reported allicin: 1.5 mg/tablet (6 mg/day); approximately 2 g fresh garlic equivalent/tablet. Other organosulfur constituents: NR.	Garlic supplementation reduced HOMA-IR, fasting insulin, and malondialdehyde, increased total antioxidant capacity and superoxide dismutase, and decreased body fat percentage and waist circumference while increasing skeletal muscle mass.
Sangouni et al. (2020b) [30]	88	45.2 ± 12.4	44.2 ± 11.1	33/12	24/19	12	Garlic powder, 400 mg/tablet, four tablets/day (1.6 g/day), vs. placebo	Reported allicin: 1.5 mg/tablet (6 mg/day); approximately 2 g fresh garlic equivalent/tablet. Other organosulfur constituents: NR.	Garlic supplementation reduced the grade of hepatic steatosis and decreased ALT, AST, GGT, total cholesterol, triglycerides, and LDL-C. HDL-C also decreased, indicating that the changes in lipid parameters were not uniformly favorable.
Soleimani et al. (2021) [31]	98	46.4 ± 11.3	44.1 ± 11.8	19/28	21/30	15	Garlic powder, 400 mg/tablet, twice daily (800 mg/day), vs. placebo	Reported allicin: 1.5 mg/tablet (3 mg/day). Other organosulfur constituents: NR.	Garlic supplementation reduced blood pressure and was associated with reduced hs-CRP.

Abbreviations: ALT, alanine aminotransferase; AST, aspartate aminotransferase; C, control; GGT, gamma-glutamyl transferase; HDL-C, high-density lipoprotein cholesterol; HOMA-IR, homeostasis model assessment of insulin resistance; hs-CRP, high-sensitivity *C*-reactive protein; LDL-C, low-density lipoprotein cholesterol; MASLD, metabolic dysfunction-associated steatotic liver disease; NR, not reported; RCTs, randomized controlled trials; SD, standard deviation; T, treatment.

**Table 2:** Representative geographical and processing-related variations in the organosulfur composition of garlic powders and powder-source garlic materials.

Geographical Region	Garlic Material/Preparation	Main Source of Compositional Variation	Reported Organosulfur Composition	Analytical Method	Reference
North America: United States	California Late garlic cultivated in Gilroy, California, and commercial garlic powder supplements marketed in the United States	Cultivar, product formulation, alliinase activity, coating, and storage; the raw-material origin of the commercial supplements was not reported	California Late garlic contained 10.8 mg/g FW alliin and generated approximately 3.9 mg/g FW allicin after homogenization. Commercial garlic powders contained 6.5–49 mg/g of alliin and showed 22–111% allicin bioavailability	HPLC and breath allyl methyl sulfide analysis	Lawson et al. (2018) [32]
South America: Argentina	Garlic powder experimentally prepared from 11 cultivars	Cultivar-related variation	Allicin yield: 8.16–17.4 mg/g DW; other organosulfur constituents NR	HPLC	Camargo et al. (2005) [33]
South Asia: India	Garlic homogenates from 93 ecotypes, reported on FW and DW bases	Ecotype and breeding-status differences	Allicin: 1.48–5.32 mg/g FW and 5.03–18.24 mg/g DW; allyl methyl thiosulfinate: 0.97–2.67 mg/g FW and 2.93–9.19 mg/g DW; allyl trans-1-propenyl thiosulfinate: 0.16–0.44 mg/g FW and 0.45–1.58 mg/g DW	UPLC-DAD	Khar et al. (2011) [34]
East Asia: China	Freeze-dried and oven-dried garlic powders	Drying method and temperature	Total thiosulfinate content was 2.2 mmol/g in freeze-dried garlic powder and decreased from 1.99 mmol/g at 40 °C to 1.1 mmol/g at 60 °C in oven-dried powders.	Spectrophotometric total thiosulfinate assay	Li et al. (2022) [35]
Europe and Africa: Poland, Portugal, Madeira, and Egypt	Powdered garlic samples prepared for compositional analysis	Geographical origin and cultivar	Polish garlic contained the highest levels of allyl organosulfur compounds, whereas Egyptian garlic showed the lowest levels of the identified organosulfur metabolites. Significant differences were also observed in amino acids, choline, sugars, and organic acids	qNMR and LC–MS	Pacholczyk-Sienicka et al. (2024) [36]
Oceania: Australia	Garlic bulbs from 32 varieties, analyzed on FW and DW bases	Genotype, mineral nutrition, and growing conditions	Allicin concentrations varied substantially among varieties, with an approximate range of 3.5–6.6 mg/g FW. Alliin and γ-glutamyl-S-allyl-L-cysteine were identified as major sulfur-containing constituents. Sulfur nutrition was associated with bulb allicin concentration	HPLC and X-ray absorption spectroscopy	Nguyen et al. (2024) [37]
Southeast Asia: Philippines	Twelve garlic accessions collected in Ilocos Norte	Accession/cultivar-related variation	Alliin: 19.78–32.84 mg/g FW; Ilocos Pink showed the highest alliin and total phenolic contents. Allicin and other thiosulfinates were not directly quantified	HPLC and Folin–Ciocalteu assay	Cava et al. (2026) [38]

Abbreviations: DW, dry weight; FW, fresh weight; HPLC, high-performance liquid chromatography; LC–MS, liquid chromatography–mass spectrometry; NR, not reported; qNMR, quantitative nuclear magnetic resonance spectroscopy; UPLC-DAD, ultra-performance liquid chromatography with diode-array detection.

**Table 3:** Characteristics of included animal studies of garlic-derived interventions in MASLD.

References	Animal	Sex	Duration	Intervention	Composition	Main Findings
Li et al. (2026) [40]	C57BL/6J mice, 4 weeks old; each group contained 8 mice	Male only	10 wk induction + 5 wk treatment	G1: normal diet; G2: HFD; G3: HFD + free allicin 10 mg/kg, i.p.; G4: HFD + liposomal allicin; G5: HFD + low-dose aptamer-functionalized liposomal allicin; G6: HFD + high-dose aptamer-functionalized liposomal allicin	Interventions delivering isolated allicin: free allicin, allicin-loaded liposomes, and aptamer-functionalized allicin-loaded liposomes. These formulations provide direct evidence for allicin, although their delivery systems differ.	Reduced body weight, hepatic steatosis, and liver injury, accompanied by downregulation of lipogenesis-related genes. Low-dose aptamer-functionalized liposomal allicin outperformed free allicin without apparent toxicity.
Deng et al. (2025) [11]	SPF C57BL/6J mice, 4 weeks old; each group contained 16 mice	Male only	8 wk	G1: normal diet + saline; G2: high-fat, high-fructose diet + saline; G3: high-fat, high-fructose diet + allicin 20 mg/kg body weight/day, orally	Isolated allicin. No other garlic-derived constituents were administered.	Allicin reduced body weight gain, hepatic inflammation, liver injury, glucose intolerance, and insulin resistance. It also partially restored hepatic Rev-erbα-related circadian rhythms, gut microbiota composition and rhythmicity, and short-chain fatty acid levels.
Sharma et al. (2025) [41]	Wistar rats; each group contained 6 rats.	NR	9 wk	G1: normal diet; G2: NASH model; G3: probiotic alone; G4: Lactobacillus plantarum and aged garlic extract model; G5: peroxisome proliferator-activated receptor α antagonist plus synbiotic model.	Complex synbiotic consisting of Lactobacillus plantarum and aged garlic extract. The organosulfur composition and allicin content of the aged garlic extract were NR.	Reduced steatosis and inflammation. Improved gut barrier function.
Liu et al. (2023) [42]	Male C57BL/6 mice, 6–8 weeks old; each group contained 10 mice.	Male only	8 wk induction + 4 wk treatment	G1: normal diet; G2: HFD + fructose + phosphate-buffered saline; G3: HFD + fructose + garlic-derived exosome-like nanoparticles 10 mg/mouse; G4: 20 mg/mouse; G5: 40 mg/mouse, gavage every 2 days.	Garlic-derived exosome-like nanoparticles; miR-396e was identified as a major functional cargo. Allicin and other organosulfur compounds were not quantified.	Reduced inflammation and hepatic lipid accumulation. Improved liver function.
Wang et al. (2023) [13]	Wistar rats, 6 weeks old; each group contained 6–8 rats.	Male only	6 wk	G1: normal diet; G2: diabetes model; G3: diabetes model + allicin 60 mg/kg/day, gavage.	Isolated allicin. No other garlic-derived constituents were administered.	Improved glucose and lipid metabolism. Modulated gut microbiota and bile acid profiles.
Yun et al. (2022) [43]	Male C57BL/6N mice, 6 weeks old; each group contained 7 mice.	Male only	5 days	G1: normal diet; G2: tunicamycin; G3: alliin 20 mg/kg/day; G4: capsaicin 20 mg/kg/day; G5: gingerol 20 mg/kg/day, gavage; tunicamycin 2 mg/kg, i.p., day 4.	Chemically defined alliin, not allicin.	Reduced steatosis, endoplasmic reticulum stress, and lipid levels.
Jiang et al. (2021) [44]	C57BL/6 mice; each group contained 10 mice.	Male only	6 wk	G1: normal diet; G2: HFD; G3: HFD + BGE 100 mg/kg/day; G4: HFD + AGE 100 mg/kg/day; G5: HFD + BGE + AGE 100 mg/kg/day; G6: 150 mg/kg/day; G7: 200 mg/kg/day, oral.	Complex combination of black ginseng extract and aged garlic extract. The organosulfur composition and allicin content of the aged garlic extract were NR.	Reduced liver injury markers. Improved glucose tolerance and insulin sensitivity.
Fajrani et al. (2021) [45]	Male Sprague Dawley rats, 150–200 g; each group contained 6 rats.	Male only	8 wk induction + 4 wk treatment	G1: healthy control; G2: NAFLD control; G3: NAFLD + metformin 45 mg/200 gBW; G4: NAFLD + vitamin E 9 IU/200 gBW; G5: NAFLD + black garlic 450 mg/200 gBW; G6: NAFLD + black garlic 900 mg/200 gBW; G7: NAFLD + black garlic 1350 mg/200 gBW.	Complex black garlic preparation. The contents of S-allyl cysteine, allicin, and other organosulfur constituents were NR.	Reduced visceral fat and oxidative stress. Improved insulin sensitivity.
Maeda et al. (2019) [46]	Male ddY-H mice, 6 weeks old; male ddY-L mice, 6 weeks old; each group contained 8 mice.	Male only	7 wk	G1: ddY-H control diet; G2: ddY-H + AGE diet 4%; G3: ddY-L control diet; G4: ddY-L + AGE diet 4%.	Complex aged garlic extract incorporated into the diet at 4% (*w*/*w*). A comprehensive organosulfur profile and allicin content were NR.	Reduced hepatic triglyceride levels in ddY-H mice. Improved insulin sensitivity and modulated gut microbiota composition.
Shi et al. (2019) [47]	C57BL/6 mice, 6 weeks old; each group contained 6 mice.	Male only	8 wk	G1: NFD; G2: HFD + saline; G3: HFD + allicin 100 mg/kg/day, oral.	Isolated allicin. No other garlic-derived constituents were administered.	Reduced adiposity. Improved glucose tolerance. Modulated gut microbiota.
Wu et al. (2015) [48]	Sprague Dawley rats, 200–250 g; each group contained 8 rats.	NR	8 wk	G1: control diet; G2: MCD; G3: MCD + antioxidant 1 100 mg/kg/day, i.p.; G4: MCD + antioxidant 2 100 mg/kg/day, i.p.; G5: MCD + silymarin 20 mg/kg/day, i.p.	Two chemically defined garlic-skin antioxidants: *N*-trans-p-coumaroyloctopamine and *N*-trans-feruloyloctopamine. These compounds are not allicin or organosulfur.	Reduced oxidative stress and liver injury markers. Improved liver histopathology.
Lai et al. (2014) [49]	C57BL/6J mice; each group contained 10 mice.	Male only	12 wk	G1: normal diet; G2: HFD; G3: HFD + GEO 25 mg/kg/day, gavage; G4: HFD + GEO 50 mg/kg/day, gavage; G5: HFD + GEO 100 mg/kg/day, gavage; G6: HFD + DADS 10 mg/kg/day, gavage; G7: HFD + DADS 20 mg/kg/day, gavage.	Garlic essential oil containing DADS (40.7%), diallyl trisulfide (21.6%), and diallyl sulfide (6.7%); DADS was also evaluated separately. Allicin was not the tested intervention.	Reduced obesity, dyslipidemia, and hepatic steatosis. Improved oxidative status.
Xiao et al. (2013a) [50]	Female SD rats, 180–200 g; each group contained 7 rats.	Female only	8 wk	G1: control diet; G2: NAFLD; G3: SAMC 200 mg/kg, i.p., three times/week; G4: NAFLD + SAMC 200 mg/kg, i.p., three times/week.	Chemically defined S-allylmercaptocysteine (SAMC), not allicin.	Reduced steatosis, liver injury, and apoptosis. Enhanced autophagy.
Takemura et al. (2013) [51]	OLETF rats and LETO rats; each group contained 5–6 rats.	Male only	13 wk	G1: LETO; G2: LETO + SAC diet 0.45%; G3: OLETF; G4: OLETF + SAC diet 0.45%.	Chemically defined S-allyl cysteine (SAC), not allicin.	Improved glycemic control and lipid metabolism. Reduced hepatic steatosis.
Xiao et al. (2013b) [52]	Female rats; each group contained 7 rats.	Female only	8 wk	G1: control diet; G2: NAFLD; G3: SAMC 200 mg/kg, i.p., three times/week; G4: NAFLD + SAMC 200 mg/kg, i.p., three times/week.	Chemically defined S-allylmercaptocysteine (SAMC), not allicin.	Reduced liver injury, lipid accumulation, fibrosis, and oxidative stress.

Abbreviations: AGE, aged garlic extract; BGE, black ginseng extract; CD36, cluster of differentiation 36; DADS, diallyl disulfide; GEO, garlic essential oil; HFD, high-fat diet; i.p., intraperitoneal injection; LETO, Long Evans Tokushima Otsuka rats; MASLD, metabolic dysfunction-associated steatotic liver disease; MCD, methionine–choline-deficient diet; NR, not reported; NAFLD, non-alcoholic fatty liver disease; NFD, normal-fat diet; OLETF, Otsuka Long Evans Tokushima Fatty rats; SAC, S-allyl cysteine; SAMC, S-allylmercaptocysteine; G1–G7 represent the experimental groups defined in the original studies.

## Data Availability

No new data were created or analyzed in this study. Data sharing is not applicable to this article.

## Abbreviations

The following abbreviations are used in this manuscript:

AMPK	AMP-activated protein kinase
ER	endoplasmic reticulum
MASLD	metabolic dysfunction-associated steatotic liver disease
PPARγ	peroxisome proliferator-activated receptor γ
RCT	randomized controlled trial
SREBP-1c	sterol regulatory element-binding protein 1c
